# Peroxisome proliferator-activated receptors γ/mitochondrial uncoupling protein 2 signaling protects against seizure-induced neuronal cell death in the hippocampus following experimental status epilepticus

**DOI:** 10.1186/1742-2094-9-184

**Published:** 2012-07-31

**Authors:** Yao-Chung Chuang, Tsu-Kung Lin, Hsuan-Ying Huang, Wen-Neng Chang, Chia-Wei Liou, Shang-Der Chen, Alice YW Chang, Samuel HH Chan

**Affiliations:** 1 Department of Neurology, Kaohsiung Chang Gung Memorial Hospital and Chang Gung University College of Medicine, Kaohsiung, 83301, Taiwan; 2 Center for Translational Research in Biomedical Sciences, Kaohsiung Chang Gung Memorial Hospital and Chang Gung University College of Medicine, Kaohsiung, 83301, Taiwan; 3 Department of Pathology, Kaohsiung Chang Gung Memorial Hospital and Chang Gung University College of Medicine, Kaohsiung, 83301, Taiwan

**Keywords:** Status epilepticus, Mitochondrial uncoupling protein 2, Peroxisome proliferator-activated receptors γ, Hippocampal neuronal cell death, Oxidative stress

## Abstract

**Background:**

Status epilepticus induces subcellular changes that may lead to neuronal cell death in the hippocampus. However, the mechanism of seizure-induced neuronal cell death remains unclear. The mitochondrial uncoupling protein 2 (UCP2) is expressed in selected regions of the brain and is emerged as an endogenous neuroprotective molecule in many neurological disorders. We evaluated the neuroprotective role of UCP2 against seizure-induced hippocampal neuronal cell death under experimental status epilepticus.

**Methods:**

In Sprague–Dawley rats, kainic acid (KA) was microinjected unilaterally into the hippocampal CA3 subfield to induce prolonged bilateral seizure activity. Oxidized protein level, translocation of Bcl-2, Bax and cytochrome *c* between cytosol and mitochondria, and expression of peroxisome proliferator-activated receptors γ (PPARγ) and UCP2 were examined in the hippocampal CA3 subfield following KA-induced status epilepticus. The effects of microinjection bilaterally into CA3 area of a PPARγ agonist, rosiglitazone or a PPARγ antagonist, GW9662 on UCP2 expression, induced superoxide anion (O_2_^· -^) production, oxidized protein level, mitochondrial respiratory chain enzyme activities, translocation of Bcl-2, Bax and cytochrome *c*, and DNA fragmentation in bilateral CA3 subfields were examined.

**Results:**

Increased oxidized proteins and mitochondrial or cytosol translocation of Bax or cytochrome *c* in the hippocampal CA3 subfield was observed 3–48 h after experimental status epilepticus. Expression of PPARγ and UCP2 increased 12–48 h after KA-induced status epilepticus. Pretreatment with rosiglitazone increased UCP2 expression, reduced protein oxidation, O_2_^· -^ overproduction and dysfunction of mitochondrial Complex I, hindered the translocation of Bax and cytochrome *c*, and reduced DNA fragmentation in the CA3 subfield. Pretreatment with GW9662 produced opposite effects.

**Conclusions:**

Activation of PPARγ upregulated mitochondrial UCP2 expression, which decreased overproduction of reactive oxygen species, improved mitochondrial Complex I dysfunction, inhibited mitochondrial translocation of Bax and prevented cytosolic release of cytochrome *c* by stabilizing the mitochondrial transmembrane potential, leading to amelioration of apoptotic neuronal cell death in the hippocampus following status epilepticus.

## Background

Epileptic seizure is a major form of acute brain damage that could lead to a large number of changes at the cellular level, including oxidative stress, cytokine activation, changes in plasticity or activation of some late cell death pathways [1,2]. In particular, prolonged and continuous epileptic seizures (status epilepticus) results in significant cerebral damage and increases the risk of subsequent epileptic episodes, along with a characteristic pattern of preferential neuronal cell loss in the hippocampus that is accompanied by long-term behavioral changes and cognitive decline [3,4]. It follows that prevention of seizure-induced hippocampal neuronal damage is also an important goal for treatment of status epilepticus. However, the cellular and molecular mechanisms via which status epilepticus induces neuronal cell death in the hippocampus remain to be fully understood.

Animal [5-8] and human [9] studies suggest that mitochondrial dysfunction occur as a consequence of prolonged epileptic seizures and may play a pivotal role in seizure-induced brain damage. Prolonged seizures affect selectively complex I in the respiratory chain; the induced oxidative and nitrosative stress precede neuronal cell death in the hippocampus and cause subsequent epileptogenesis [2,10]. Therefore, the mitochondria can be considered a target for potential neuroprotective strategies in epilepsy.

The uncoupling proteins (UCPs) have emerged as important natural antioxidants in the maintenance of reactive oxygen species (ROS) homeostasis [11]. UCPs belong to a superfamily of mitochondrial anion transporters that uncouple ATP synthesis from oxidative phosphorylation by causing proton leakage across the mitochondrial inner membrane, leading to energy dissipation and heat production [12]. More importantly, the resultant decrease in proton electrochemical gradient across the inner mitochondrial membrane elicited by the UCPs mitigates mitochondrial ROS production [11,13]. In mammals, five homologues, *UCP1* to *UCP5*, have so far been cloned [13]. Among them, accumulating evidence suggests that an increase in *UCP2* gene expression is related to the decline of mitochondrial ROS production [14,15]. UCP2 has been widely studied in the context of obesity, diabetes mellitus and inflammatory responses [14,16]; an absence of UCP2 potentially promotes ROS accumulation and induces oxidative damages and inflammatory response. In the central nervous system (CNS), UCP2 has been shown to be upregulated by stress signals such as kainate administration, injury or ischemia, and overexpression of UCP2 has been reported to be neuroprotective against oxidative stress *in vivo* and *in vitro*[13,17,18]. However, the exact mechanism has not been fully established.

We have shown previously that dysfunction of complex I respiratory chain enzyme and mitochondrial ultrastructural damage in the hippocampus are associated with prolonged seizure during experimental temporal lobe status epilepticus [5]. Based on this animal model, our recent studies [19-22] demonstrated that an excessive production of nitric oxide (NO) generated by the upregulated NO synthase II (NOS II), accompanied by an increase in superoxide anion (O_2_^·-^) production and peroxynitrite formation, followed by a reduction in mitochondrial complex I activity and release of cytochrome *c* from mitochondria to the cytosol, which triggers the caspase cascades that lead to apoptotic cell death in the hippocampus. In addition to this detrimental chain reaction under status epilepticus, it is conceivable that cellular responses that counteract these detrimental effects may be activated as an endogenous protective mechanism. In this regard, we have demonstrated previously that rosiglitazone, a peroxisome proliferator-activated receptor γ (PPARγ) agonist, enhances UCP2 expression after cerebral ischemia to protect against neuronal cell death in the hippocampus [23,24]. It follows that as an antioxidant, UCP2 may be activated during experimental status epilepticus, leading to decreased ROS production, reduced mitochondrial dysfunction, impeded apoptotic pathway and retarded neuronal injury in the hippocampus. Results from the present study validated this hypothesis.

## Methods

All experimental procedures were carried out in compliance with the guidelines for the care and use of experimental animals endorsed by our institutional animal care committee. All efforts were made to reduce the number of animals used and to minimize animal suffering during the experiment.

### Animals

Experiments were carried out in specific pathogen-free adult male Sprague–Dawley rats (260 to 300 g) that were obtained from the Experimental Animal Center of the National Science Council, Taiwan, Republic of China. They were housed in an animal room under temperature control (24 to 25°C) and a 12-h light–dark (08:00 to 20:00 h) cycle. Standard laboratory rat chow and tap water were available *ad libitum*.

### Experimental temporal lobe status epilepticus

An experimental model of temporal lobe status epilepticus established previously by us [5,19-21] was used. This model entails microinjection unilaterally of kainic acid (KA) into the hippocampal CA3 subfield that results in a progressive buildup of bilateral seizure-like hippocampal electroencephalographic (hEEG) activity. The head of the animal was fixed to a stereotaxic headholder (Kopf, Tujunga, CA, USA) after intraperitoneal (ip) administration of chloral hydrate (400 mg/kg) to induce anesthesia, and the rest of the body was placed on a heating pad to maintain body temperature at 37°C. KA (0.5 nmol; Tocris Cookson, Bristol, UK) dissolved in 0.1 M PBS, pH 7.4, was microinjected stereotaxically (3.3 to 3.6 mm posterior to the bregma, 2.4 to 2.7 mm from the midline, and 3.4 to 3.8 mm below the cortical surface) into the CA3 subfield of the hippocampus on the left side. The volume of microinjection of KA was restricted to 50 nL and was delivered using a 27-gauge needle connected to a 0.5-μL Hamilton microsyringe (Reno, NV, USA). This consistently resulted in progressive and concomitant increase in both root mean square and mean power frequency values of hEEG signals recorded from the CA3 subfield on the right side [5,20,21]. As a routine, these experimental manifestations of continuous seizure activity were followed by hEEG for 60 minutes, followed by ip administration of diazepam (30 mg/kg) to terminate seizures [5,20,21]. The wound was then closed in layers, and sodium penicillin (10,000 IU; YF Chemical Corporation, Taipei, Taiwan) was given intramuscularly to prevent postoperative infection. Animals were returned to the animal room for recovery in individual cages. Rats that received unilateral microinjection of 50 nL of PBS and did not exhibit seizure-like hEEG activities served as our vehicle controls. Animals that received choral hydrate anesthesia and surgical preparations without additional experimental manipulations served as sham-controls.

### Pharmacological pretreatments

In experiments that involved pharmacological pretreatments, test agents were microinjected bilaterally and sequentially into the CA3 subfield of the hippocampus, at a volume of 150 nL on each side. Test agents used included an activator of PPARγ [24,25], rosiglitazone (Cayman Chemical, Ann Arbor, MI, USA) and a PPARγ antagonist [25,26], GW9662 (Cayman Chemical). The doses of test agents used were 6 nmol for rosiglitazone, and 500 ng for GW9662 [24,26]. Microinjection of 3% dimethyl sulfoxide (DMSO) solvent served as the vehicle and volume control. To avoid the confounding effects of drug interaction, each animal received only one single pharmacological pretreatment, followed 30 minutes later by microinjection of KA (0.5 nmol) or PBS into the left hippocampal CA3 subfield.

### Collection of tissue samples from the hippocampus

At predetermined time intervals (3, 6, 12, 24 or 48 h; or 7 days) after microinjection of KA or PBS into the hippocampus, rats were again anesthetized by ip administration of chloral hydrate (400 mg/kg,) and were perfused intracardially with 50 mL of warm (37°C) saline that contained heparin (100 U/mL). As we reported previously [5,19-21], the brain was rapidly removed under visual inspection and placed on a piece of gauze moistened with ice-cold 0.9% saline. We routinely collected tissues from the ipsilateral (injection side for KA) and the contralateral (recording side for hEEG) hippocampal CA3 subfield [5,19-21]. Hippocampal samples were stored at −80°C until biochemical analysis. In experiments involving immunofluorescence staining, brains were post-fixed in 4% paraformaldehyde for 48 h at 4°C followed by cryoprotection with 30% sucrose solution. The concentration of total proteins extracted from tissue samples was determined by the bicinchoninic acid (BCA) protein assay (Pierce, Rockford, IL, USA). In some experiments, proteins from the nuclear or cytosolic fraction of the hippocampal samples were extracted by a commercial kit (Active Motif, Carlsbad, CA, USA).

### Measurement of protein oxidation

Oxidized protein was determined using a protein oxidation detection kit (OxyBlot, Chemicon, Temecula, CA, USA). This kit provides reagents for sensitive immunodetection of the carbonyl group, which is a hallmark of the oxidation status of proteins [27,28]. Total proteins extracted from the hippocampal CA3 subfield were reacted with 2,4-dinitrophenylhydrazine and derivatized to 2,4-dinitrophenylhydrazone (DNP-hydrazone) [29]. The DNP-derivatized protein samples were separated on a 15% SDS-polyacrylamide gel followed by western blot. The blot was incubated with a rabbit anti-DNP antibody, followed by incubation with a horseradish peroxidase-conjugated goat anti-rabbit IgG according to manufacturers’ instructions.

### Western blot analysis

Western blot analysis for UCP2, PPARγ, Bcl-2, Bax, cytochrome *c* or β-actin was carried out on proteins extracted from nuclear fractions or from mitochondrial or cytosolic fractions of hippocampal samples. The purity of the mitochondrial fraction was verified by the selective expression of the mitochondrial inner membrane-specific protein, cytochrome *c* oxidase subunit IV (COX IV). Protein concentration was determined by the BCA Protein Assay (Pierce). The primary antisera used included rabbit polyclonal antiserum against Bax and COX IV (Cell Signaling, Danvers, MA, USA), goat polyclonal antiserum against UCP2 (Santa Cruz Biotechnology, Santa Cruz, CA, USA), mouse monoclonal antiserum against Bcl-2, cytochrome *c* and PPARγ (Santa Cruz Biotechnology) or β-actin (Chemicon, Temecula, CA, USA). β-actin was used for internal control of total protein or proteins in the cytosolic fraction, and COX IV for proteins in the mitochondrial fraction. . The secondary antisera used included horseradish peroxidase-conjugated sheep anti-mouse IgG (Amersham Biosciences, Little Chalfont, UK) for Bcl-2, cytochrome *c*, PPARγ and β-actin; donkey anti-goat IgG (Chemicon) for UCP2, or donkey anti-rabbit IgG (Amersham Biosciences) for Bax, and COX IV. Specific antibody-antigen complex was detected by an enhanced chemiluminescence western blot detection system (NEN, Boston, MA, USA). The amount of protein was quantified by ImageMaster software (Amersham Pharmacia Biotech, Piscataway, NJ, USA), and was expressed as the ratio relative to β-actin protein (for analysis of total protein or proteins in the cytosolic fraction) or COX IV (for analysis of proteins in the mitochondrial fraction).

### RNA isolation and reverse transcription real-time polymerase chain reaction

For quantitative analysis of *Ucp2* mRNA expression in the hippocampal CA3, at 3, 6, 12 h or 24 h after microinjection of KA or PBS into the hippocampus, the brain was rapidly removed and total RNA from the hippocampal CA3 was isolated with TRIzol reagent (Invitrogen) according to the manufacturer’s protocol. All RNA isolated was quantified by spectrophotometry and the optical density 260/280 nm ratio was determined. RT reaction was performed using a SuperScript Preamplification System (Invitrogen) for the first-strand cDNA synthesis [25,30]. Real-time PCR for amplification of cDNA was performed using a LightCycler (Roche Diagnostics, Mannheim, Germany). PCR for each sample was carried out in duplicate for all cDNAs and for the glyceraldehyde-3-phosphate dehydrogenase (GAPDH) control [25,30]. The PCR mixture (total volume 20 μl), which was prepared with nuclease-free water, contained 2 μL of LightCycler FastStart DNA Master SYBR Green 1 (Roche Diagnostics), 3 mM MgCl2, and 5 μM each primer, together with 5 μL of purified DNA or negative control. The primer pairs for amplifi-cation of *Ucp2* cDNA (GenBank: U69135) were 5′-TCCCCTGTTGATGTGGTCAA-3′ for the forward primer, and 5′-CAGTGACCTGCGCTGTGGTA-3′ for the reverse [25]. Primer pairs for GAPDH cDNA (GenBank: NM017008) were 5′-GCCAAAAGGGTCATCATCTC-3′ for the forward primer, and 5′-GGCCATCCACAGTCTTCT-3′ for the reverse [25]. The amplification protocol for cDNA was a 10-minute denaturation step at 95°C for polymerase activation; a so-called touchdown PCR step of 10 cycles consisting of 10 s at 95°C, 10s at 65°C, and 30s at 72°C; followed by 40 cycles consisting of 10 s at 95°C, 10 s at 55°C, and 30s at 72°C. After slow heating (0.1°C per second) of the amplified product from 65 to 95°C to generate a melting temperature curve, which serves as a specificity control, the PCR samples were cooled to 40°C. The PCR products were subsequently subjected to agarose gel electrophoresis for further confirmation of amplification specificity. Fluorescence signals from the amplified products were quantitatively assessed using the LightCycler software program (version 3.5). Second derivative maximum mode was chosen with baseline adjustment set in the arithmetic mode. The relative change in *Ucp2* mRNA expression was determined by the fold-change analysis [25], in which:

Fold change = 2^−(ΔΔC*t*)^

where:

(1) $$\Delta \Delta \text{C}t={\left(\text{C}t_{\text{UCP}2}-\text{C}t_{\text{GAPDH}}\right)}_{\text{pharmacological treatment}}-{\left(\text{C}t_{\text{UCP}2}-\text{C}t_{\text{GAPDH}}\right)}_{\text{control}})$$

Note that C*t* value is the cycle number at which the fluorescence signal crosses the threshold.

### Double immunofluorescence staining and laser confocal microscopy

Free-floating sections of the hippocampus (35 μm) were processed for double immunofluorescence staining by procedures we reported previously [19-21]. Double immunofluorescence staining was carried out using a rabbit polyclonal antiserum against UCP2 (Bioss, Woburn, MA, USA) or against a marker for astrocytes, glial fibrillary acidic protein (GFAP; Abcam, Cambridge, UK); or rabbit polyclonal antiserum against a mitochondrial membrane protein, COX IV (Cell Signaling). The secondary antisera included a goat anti-rabbit IgG conjugated with AlexaFluor 488 and a goat anti-mouse IgG conjugated with Alexa Fluor 568 or a goat anti-rabbit IgG conjugated with AlexaFluor 546 (Molecular Probes, Eugene, OR, USA). Hippocampal CA3b area was viewed under a Fluorview FV10i laser scanning confocal microscope (Olympus, Tokyo, Japan), and immunoreactivity for NeuN, COX IV or GFAP exhibited red fluorescence and UCP2 manifested green fluorescence. The exhibition of yellow fluorescence on merged images indicated the presence of UCP2 immunoreactivity in neurons, mitochondria or astrocytes.

### Measurement of superoxide anion production

Measurement of O_2_^·-^ production was determined by lucigenin-enhanced chemiluminescence [21,31]. Fresh samples from the hippocampal CA3 subfield were homogenized in 20 mM sodium phosphate buffer, pH 7.4, containing 0.01 mM EDTA by a glass-to-glass homogenizer. The homogenate was subject to centrifugation at 1000 *g* for 10 minutes at 4 °C to remove nuclei and unbroken cell debris. The pellet was discarded and the supernatant was obtained immediately for O_2_^·-^ measurement. Background chemiluminescence in buffer (2 mL) containing lucigenin (5 μM) was measured for 5 minutes. An aliquot of 100 μl of supernatant was then added, and the chemiluminescence measured for 30 minutes at room temperature with a Sirius luminometer (Berthold, Germany) [21]. O_2_^·-^production was calculated and expressed as mean light units per minute per mg protein. Specificity for O_2_^·-^ was determined by adding superoxide dismutase (SOD) (350 U/mL) into the incubation medium.

### Assays for activity of mitochondrial respiratory enzymes

Isolation of rat mitochondria from the hippocampal samples was carried out according to our previous report [5,21] and modification [32]. Hippocampal tissues were suspended in wash buffer and homogenized in an ice-cold mitochondrial isolation buffer kit (MS850, MitoSciences, Eugene, OR, USA) using a loose-fit 2 mL glass homogenizer (Kontes, Vineland, NJ, USA). The homogenate was centrifuged at 1000 *g* for 10 minutes at 4°C, and the supernatant obtained was further centrifuged at 12000 *g* for 15 minutes. The pellet was resuspended in isolation buffer and protease inhibitor was added, and then centrifuged at 12000 g for 15 minutes. The final mitochondrial pellet was suspended in a minimal amount of isolation buffer and protease inhibitor and stored at −80°C until measurement of mitochondrial respiratory enzyme activity, which was undertaken within 3 days. Total protein in the mitochondrial suspension was determined by the BCA Protein Assay (Pierce, Rockford, IL, USA), using bovine serum albumin as a standard.

The activity of complex I (nicotinamide adenine dinucleotide (NADH) ubiquinone oxidoreductase) and complex IV (cytochrome *c* oxidase) of mitochondrial respiratory enzymes were analyzed by enzyme activity immunocapture assays [32,33]. A 96-well plate coated with monoclonal antibodies against the oxidative phosphorylation complex I (MS141, MitoSciences) and IV (MS444, MitoSciences) were used according to manufacturers’ instructions. Complex I activity was measured by adding an assay solution and the oxidation of NADH was monitored by measuring its decrease in absorbance at 450 nm in kinetic mode at 30°C for 2 h. Complex IV activity was measured by adding an assay solution and the oxidation of reduced cytochrome *c* was monitored by measuring its decrease in absorbance at 550 nm in kinetic mode at room temperature for 30 minutes. Assays for mitochondrial respiratory enzyme activity were performed using a Multiskan Spectrum reader (Thermo Scientific, Miami, USA). At least duplicate determination was carried out on each tissue sample.

### Qualitative and quantitative analysis of DNA fragmentation

Tissue samples from the hippocampal CA3 subfield were subject to qualitative and quantitative analysis of DNA fragmentation. After extraction of total DNA from hippocampal tissues, nucleosomal DNA ladders were amplified by a PCR kit for DNA ladder assay (Maxim Biotech, San Francisco, CA, USA) to enhance the detection sensitivity, and were separated by electrophoresis on 1% agarose gel [19-21]. To quantify apoptosis-related DNA fragmentation, a cell death ELISA (Roche Molecular Biochemicals, Mannheim, Germany) that detects apoptotic but not necrotic cell death [34] was used to assay the level of histone-associated DNA fragments in the cytoplasm [35]. Proteins from the cytosolic fraction of hippocampal samples were used as the antigen source, together with primary anti-histone antibody and secondary anti-DNA antibody coupled to peroxidase. The amount of nucleosomes in the cytoplasm was quantitatively determined using 2,2′-azino-di-[3-ethylbenzthiazoline] sulfonate as the substrate. Absorbance was measured at 405 nm and referenced at 490 nm using a microtiter plate reader (Anthros Labtec).

### Statistical analysis

One-way analysis of variance (ANOVA) was used, as appropriate, to assess group means, followed by the Scheffé multiple-range test for post hoc assessment of individual means. All values are expressed as mean ± standard error of the mean (SEM). A *P*-value < 0.05 was taken to indicate statistical significance.

## Results

### Strategies for biochemical analyses and pharmacological treatments

As in our previous studies [5,19-22], we routinely carried out biochemical analysis separately on tissues collected from the ipsilateral (injection side for KA) and the contralateral (recording side for hEEG) hippocampal CA3 subfield. This allowed us to ascertain that results from those analyses were consequential directly to experimental temporal lobe status epilepticus and not indirectly to KA excitotoxicity. Since seizure activity was activated bilaterally, test agents were also routinely microinjected into the bilateral hippocampal CA3 subfield to confirm that parallel results were obtained from CA3 areas on both sides.

### Temporal changes in protein oxidation in the hippocampal CA3 subfield following experimental temporal lobe status epilepticus

**Figure 1:**
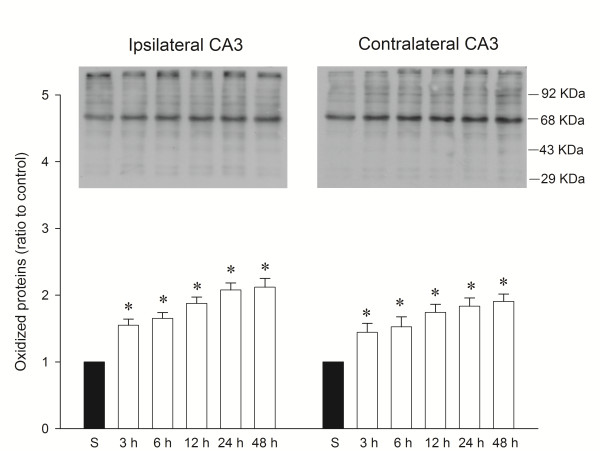
**Representative gels (inset) or temporal changes of protein oxidation detected in samples collected from the CA3 subfield of the hippocampus at 3, 6, 12, 24 and 48 h after microinjection of 0.5 nmol kainic acid (KA) or PBS into the left hippocampal CA3 subfield.** Total proteins were extracted from the hippocampal CA3 subfield at the indicted times or from sham-operated controls followed by immunoblot analysis for the extent of protein oxidation. Values in the lower panel are fold changes with reference to sham-control (S) and are mean ± standard error of the mean (SEM) of four animals per experimental group. * P  < 0.05 versus sham-control group in the Scheffé multiple-range test.

We reported recently [21] that a significant surge in O_2_^·-^ production took place as early as 3 h after the induction of experimental temporal lobe status epilepticus, which gradually declined over 24 h. Our first series of experiments therefore, established that oxidative stress damages occurred in hippocampal CA3 neural cells following experimental lobe status epilepticus. We observed a significantly heightened content of oxidized proteins in the ipsilateral hippocampal CA3 subfield as early as 3 h, followed by a progressive reduction over 24 h after unilateral microinjection of KA (0.5 nmol) into the left CA3 area (Figure 1). We also observed a significant increase in oxidized proteins in the contralateral CA3 subfield over the same time intervals after local application of KA into the left hippocampal CA3 subfield. Importantly, the temporal changes of protein oxidations in the bilateral hippocampal CA3 subfield paralleled the time course of O_2_^·-^ production after experimental status epilepticus [21].

### Temporal course of Bax and cytochrome *c* translocation in the hippocampal CA3 subfield following experimental temporal lobe status epilepticus

**Figure 2:**
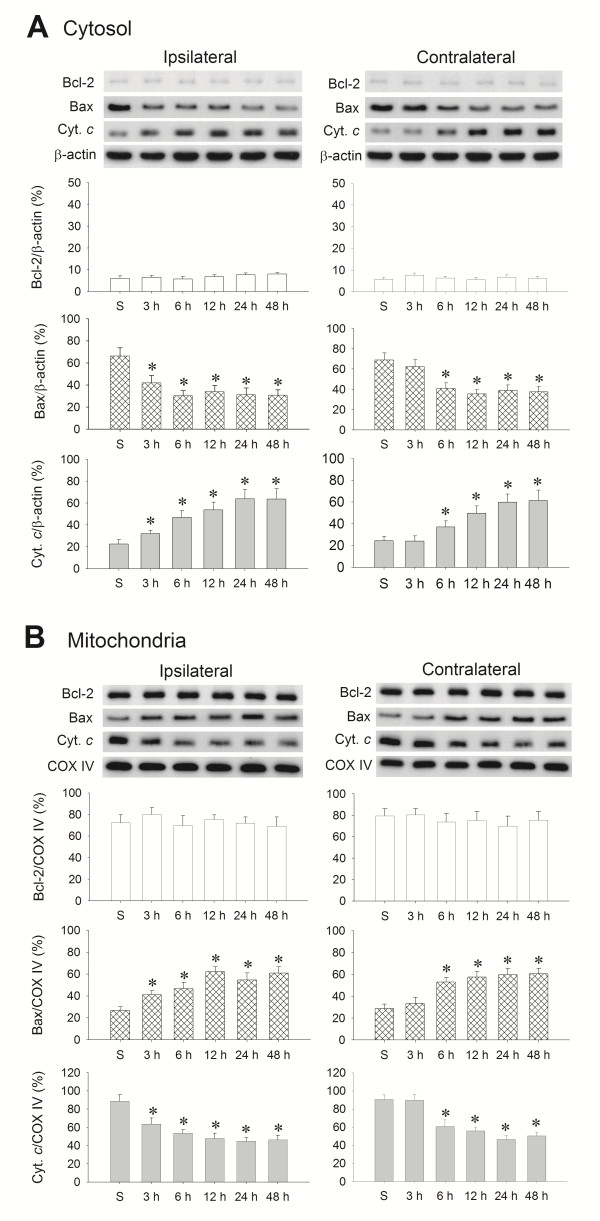
**Representative gels (inset) or temporal changes in Bcl-2, Bax or cytochrome* c *relative to β-actin protein detected in the cytosolic fraction or relative to cytochrome* c *oxidase subunit IV (COX-IV) in the mitochondrial fraction of samples collected from the CA3 subfield of the hippocampus after microinjection of kainic acid (KA) or PBS. (A)** Cytosolic fraction and **(B)** mitochondrial fraction of samples collected 3, 6, 12, 24 and 48 h after microinjection of KA (0.5 nmol) or PBS into the left hippocampal CA3 subfield. β-actin or COX-IV was used as the internal loading control for the cytosolic or mitochondrial fraction. Values are mean ± standard error of the mean (SEM) of the ratio of Bcl-2, Bax or cytochrome *c* to the loading controls, and are quadruplicate analyses from six animals per experimental group. *P < 0.05 versus sham-control (S) group in the Scheffé multiple-range test.

Our second series of experiments investigated whether the Bcl-2, Bax and cytochrome *c* signaling cascades are associated with excessive ROS production in the hippocampal CA3 subfield following experimental temporal lobe status epilepticus. Western blot analysis revealed that Bcl-2 was not discernibly altered in either the mitochondrial or cytosolic fraction of samples obtained from the hippocampal CA3 subfield. However, there was a significant decrease of Bax level and increase of cytochrome *c* level in the cytosolic fraction (Figure 2A) of samples from the bilateral hippocampal CA3 subfield after unilateral microinjection of KA (0.5 nmol) into the left CA3 region, accompanied by a corresponding increase of Bax level and decrease of cytochrome *c* level in the mitochondrial fraction (Figure 2B). Of note is that this induced mitochondria-bound translocation of Bax from the cytosol and cytosol-bound translocation of cytochrome *c* from the mitochondria followed a time frame that started from 3 h in the ipsilateral and 6 h in the contralateral CA3 area, and was sustained 48 h after the induction of experimental temporal lobe status epilepticus.

### Temporal changes of PPARγ and UCP2 expression in the hippocampal CA3 subfield following experimental temporal lobe status epilepticus

**Figure 3:**
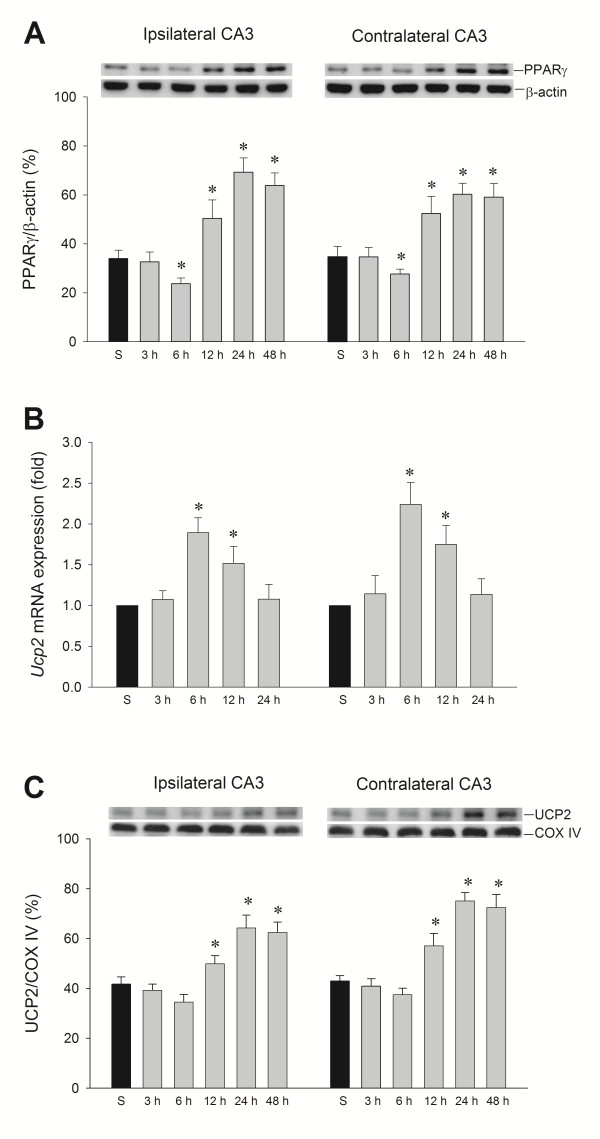
**Temporal changes in peroxisome proliferator-activated receptor γ (PPARγ) protein relative to β-actin protein and UCP2 protein relative to cytochrome*c*oxidase subunit IV (COX-IV), and real-time PCR analysis showing fold changes in uncoupling protein 2 (*Ucp2*) mRNA expression relative to glyceraldehyde-3-phosphate dehydrogenase after microinjection of kainic acid or PBS into the left hippocampal CA3 subfield. (A)** Changes in PPARγ protein relative to β-actin protein. **(B)** Fold changes in *Ucp2* mRNA expression relative to glyceraldehyde-3-phosphate dehydrogenase. **(C)** Changes in UCP2 protein relative to COX-IV. Changes were detected in samples collected from the CA3 subfield of the hippocampus at 3, 6, 12, 24 or 48 h after microinjection of 0.5 nmol kainic acid or PBS into the left hippocampal CA3 subfield. Values are mean ± standard error of the mean (SEM) of quadruplicate analyses from six animals per experimental group. *P < 0.05 versus sham-control group (S) in the Scheffé multiple-range test.

Our third series of experiments examined whether PPARγ and UCP2 in the hippocampal CA3 subfield exhibit changes in expression level following experimental temporal lobe status epilepticus. After unilateral microinjection of KA into the left CA3 region, western blot analysis revealed a slight decrease of PPARγ 6 h after ipsilateral KA treatment, followed by a significant increase of expression from 12 to 48 h in the bilateral hippocampal CA3 subfields (Figure 3A). More intriguingly, real-time PCR analysis (Figure 3B) revealed that *Ucp2* mRNA underwent a significant increase in the bilateral hippocampal CA3 area that peaked at 6 h after the elicitation of sustained hippocampal seizure discharges. Also, western blot analysis showed a significant increase of UCP2 protein expression from 12 to 48 h in both hippocampal CA3 subfields (Figure 3C) that paralleled the augmented expression of PPARγ.

### Effects of rosiglitazone and GW9662 on UCP2 expression in hippocampal CA3 neurons following experimental temporal lobe status epilepticus

**Figure 4:**
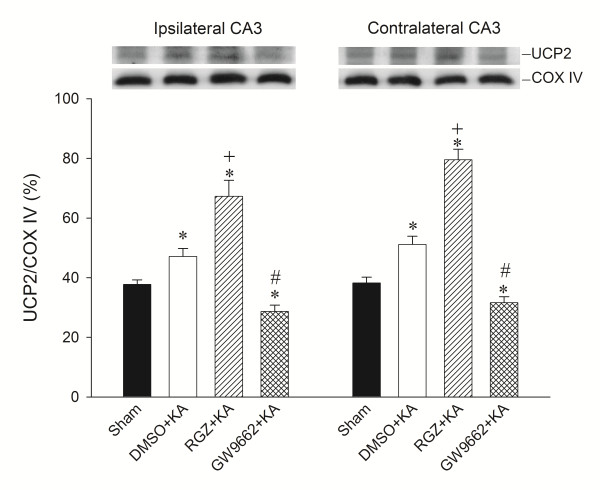
**Representative gels (inset) or changes in uncoupling protein (UCP)-2 relative to cytochrome*c*oxidase subunit IV (COX-IV), detected in the mitochondrial fraction of samples collected from the CA3 subfield of the hippocampus 24 h after microinjection of 0.5 nmol kainic acid or PBS into the left hippocampal CA3 subfield after pretreatment with 3% dimethyl sulfoxide (DMSO), rosiglitazone (RGZ, 6 nmol) or 500 ng GW9662 applied tothe bilateral CA3 subfield.** Values are mean ± standard error of the mean (SEM) of quadruplicate analyses from six animals per experimental group. *P < 0.05 versus sham-control group, +*P* < 0.05 versus sham-control, DMSO + KA or GW9662 + KA group, and #*P* < 0.05 versus sham-control, DMSO + KA or RGZ + KA group in the Scheffé multiple-range test.

**Figure 5:**
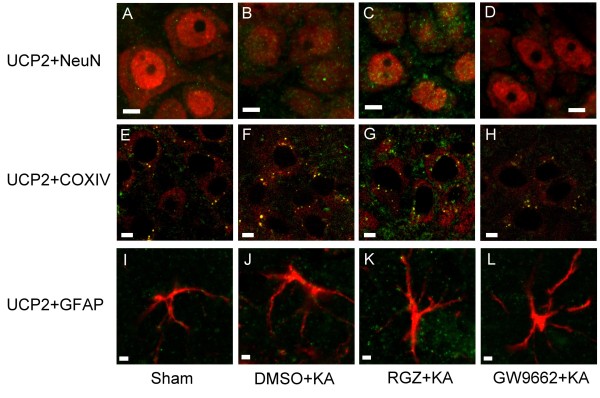
**Laser scanning confocal microscopic images of the right CA3b subregion of hippocampus showing cells that were immunoreactive to a neuronal marker, a mitochondrial protein marker, or a marker for astrocytes, and additionally stained for uncoupling protein (UCP)-2.** Scanning was performed 24 h after microinjection of 0.5 nmol kainic acid (KA) or PBS into the left hippocampal CA3 subfield in animals that received pretreatment with application into the bilateral CA3 subfield of 3% dimethyl sulfoxide (DMSO), 6 nmol rosiglitazone (RGZ) or 500 ng GW9662. **(A-D)** Neuronal marker, NeuN (red fluorescence).** (E-H)** Mitochondrial protein marker, cytochrome *c* oxidase subunit IV (COX IV) (red fluorescence). **(I-L)** Marker for astrocytes, glial fibrillary acidic protein (GFAP) (red fluorescence). Cells were additionally stained for UCP2 (green fluorescence). Note that double-labeled neurons, mitochondria or astrocytes displayed yellow fluorescence. These results are typical of three animals from each experimental group. Scale bar, 5 μm in 5A-H; 2 μm in 5I-L.

Our fourth series of experiments further explored a causal role for PPARγ and UCP2 in experimental temporal lobe status epilepticus. Bilateral microinjection of the PPARγ agonist, rosiglitazone (6 nmol) into the hippocampal CA3 region significantly increased the expression of UCP2 in the mitochondrial fraction (Figure 4) from the CA3 subfield 24 h after the elicitation of sustained hippocampal seizure discharges. On the other hand, bilateral microinjection of the PPARγ antagonist, GW9662 (500 ng) reduced the elicited UCP2 expression (Figure 4). Similar observations were obtained from double immunofluorescence staining coupled with laser scanning confocal microscopy. Compared to sham-control (Figure 5A), there was an increase in UCP2 immunoreactivity in neurons from the hippocampal CA3 subfield on the right side (Figure 5B) 24 h after KA-induced status epilepticus. Moreover, whereas pretreatment with rosiglitazone (6 nmol) increased (Figure 5C), GW9662 (500 ng) pretreatment decreased (Figure 5D) UCP2 immunoreactivity in the hippocampal CA3 neurons.

We also verified the localization of UCP2 immunoreactivity in mitochondria by co-immunofluorescence staining with the mitochondrial membrane protein, COX IV (Figure 5E-H) of hippocampal CA3 neurons on the right side (Figure 5F), 24 h after KA-induced status epilepticus compared with sham-control (Figure 5E). Additionally, pretreatment with rosiglitazone (6 nmol) increased (Figure 5G), and GW9662 (500 ng) pretreatment decreased (Figure 5H) UCP2 immunoreactivity in the mitochondria of hippocampal CA3 neurons. However, the immunoreactivity for UCP2 was not significantly changed in hippocampal cells that were immunoreactive to the astrocyte marker GFAP 24 h following experimental status epilepticus (Figure 5I-L).

### Effects of rosiglitazone and GW9662 on superoxide production and oxidized protein expression in the hippocampal CA3 subfield following experimental temporal lobe status epilepticus

**Figure 6:**
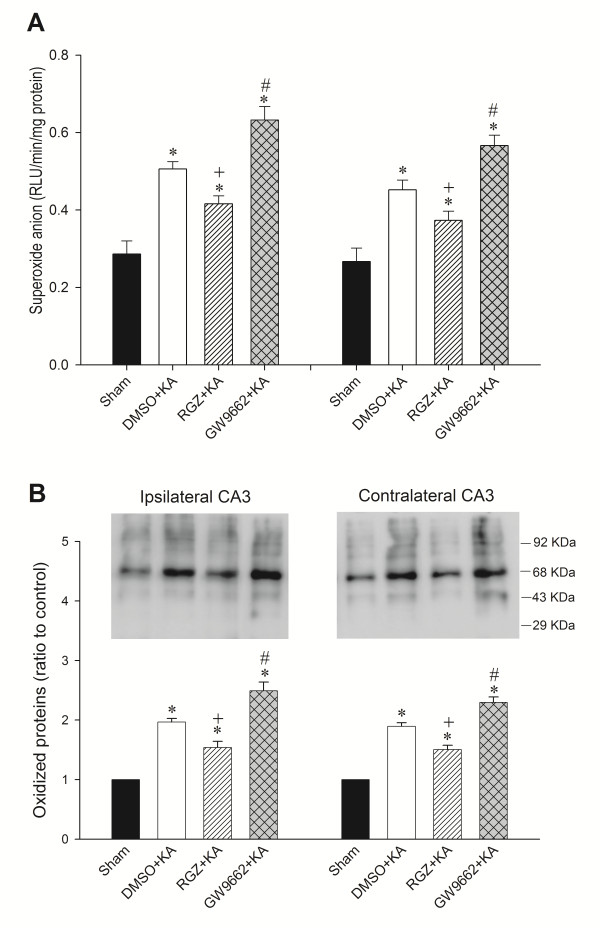
**Representative changes in superoxide anion (O**_**2**_^**·-**^**) production and protein oxidation in the bilateral CA3 subfield of the hippocampus 24 h after microinjection of kainic acid (KA) (0.5 nmol) or PBS into the left hippocampal CA3 subfield in animals that received pretreatment with application into the bilateral CA3 subfield of 3% dimethyl sulfoxide(DMSO), 6 nmol rosiglitazone (RGZ) or 500 ng GW9662. (A)** Changes in O_2_^·-^ production. **(B)** Changes in protein oxidation. Values are mean ± standard error of the mean (SEM) of quadruplicate analyses from four animals per experimental group. *P < 0.05 versus sham-control group, +*P* < 0.05 versus sham-control, DMSO + KA or GW9662 + KA group, and #*P* < 0.05 versus sham-control, DMSO + KA or RGZ + KA group in the Scheffé multiple-range test.

To strengthen a pivotal role of the PPARγ/UCP2 signaling pathway in oxidative stress damage in the hippocampus following experimental status epilepticus, we observed that bilateral microinjection of rosiglitazone into the hippocampal CA3 region, at a dose (6 nmol) that enhanced UCP2 expression, also decreased the levels of O_2_^·-^ ((Figure 6A) or oxidized protein (Figure 6B) in the CA3 subfield 24 h after KA-induced experimental status epilepticus. On the other hand, pretreatment with GW9662 (500 ng) increased the levels of O_2_^·-^ (Figure 6A) or oxidized protein (Figure 6B).

### Effects of rosiglitazone and GW9662 on the activity of mitochondrial respiratory enzymes in the hippocampal CA3 subfield following experimental temporal lobe status epilepticus

**Figure 7:**
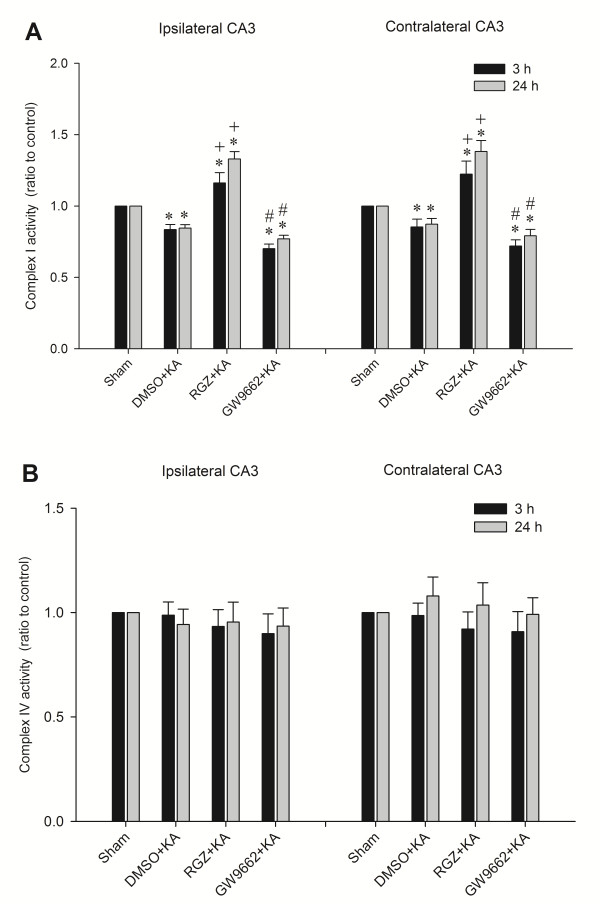
**Enzyme assay for the activity of complex I or complex IV in mitochondria isolated from the CA3 subfield of the hippocampus 3 h and 24 h after microinjection of kainic acid (KA) (0.5 nmol) or PBS into the left hippocampal CA3 subfield, in animals that received pretreatment with application into the bilateral CA3 subfield of 3% dimethyl sulfoxide (DMSO), 6 nmol rosiglitazone (RGZ) or 500 ng GW9662. (A)** Complex I. **(B)** Complex IV. Values are fold changes with reference to sham-control and are mean ± standard error of the mean (SEM) of four animals per experimental group. *P < 0.05 versus sham-control group, +*P* < 0.05 versus sham-control, DMSO + KA or GW9662 + KA group, and #*P* < 0.05 versus sham-control, DMSO + KA or RGZ + KA group in the Scheffé multiple-range test.

Our laboratory reported previously [5,21] that depression of mitochondrial complex I and preservation of complex IV enzyme activity in the hippocampus takes place in our experimental model of temporal lobe status epilepticus. Our next series of experiments examined whether the induced mitochondrial dysfunction is causally related to upregulation of UCP2. We found that the significantly reduced complex I respiratory enzyme activity in the bilateral hippocampal CA3 subfield 3 and 24 after local application of KA into the left CA3 subfield was significantly blunted by pretreatment with rosiglitazone (6 nmol) (Figure 7A). However, the induced dysfunction of complex I was aggravated by pretreatment with GW9662 (500 ng) (Figure 7A). On the other hand, there was a lack of discernible changes in complex IV activities 3 and 24 h after experimental status epilepticus in animals pretreated with rosiglitazone or GW9662 (Figure 7B).

### Effects of rosiglitazone and GW9662 on apoptotic cell death in the hippocampal CA3 subfield following experimental temporal lobe status epilepticus

**Figure 8:**
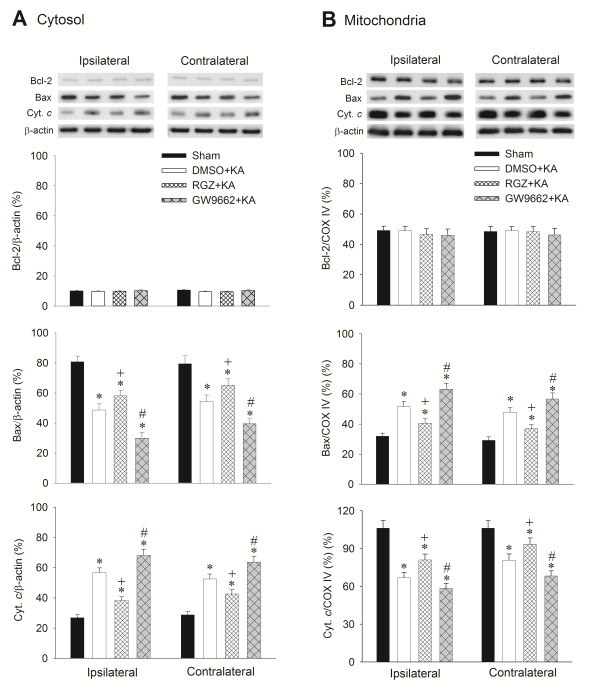
**Representative gels (inset) or temporal changes in Bcl-2, Bax or cytochrome*c*relative to β-actin protein detected in the cytosolic or relative to cytochrome*c*oxidase subunit IV (COX-IV) in the mitochondrial fraction of samples collected from the CA3 subfield of the hippocampus 24 h after microinjection of kainic acid (KA) (0.5 nmol) or PBS into the left hippocampal CA3 subfield, in animals that received pretreatment with application into the bilateral CA3 subfield of 3% dimethyl sulfoxide (DMSO), 6 nmol rosiglitazone (RGZ) or 500 ng GW9662. (A)** Cytosolic fraction and **(B)** mitochondrial fraction of samples. Values are mean ± standard error of the mean (SEM) of quadruplicate analyses from six animals per experimental group. *P < 0.05 versus sham-control group, +*P* < 0.05 versus sham-control, DMSO + KA or GW9662 + KA group, and #*P* < 0.05 versus sham-control, DMSO + KA or RGZ + KA group in the Scheffé multiple-range test.

**Figure 9:**
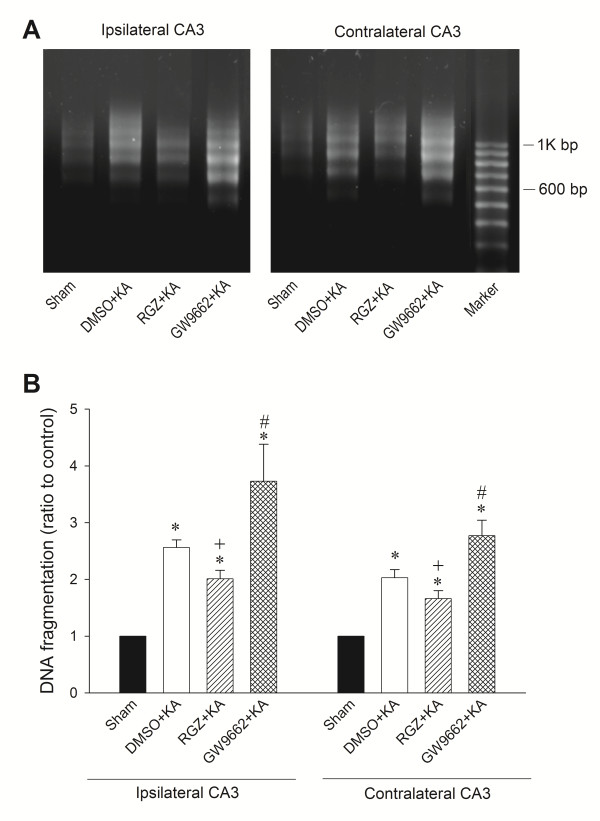
**Analysis of DNA fragmentation detected in samples collected from the CA3 subfield of the hippocampus 7 days after microinjection of kainic acid (KA) (0.5 nmol) or PBS into the left hippocampal CA3 subfield in animals that received pretreatment with application into the bilateral CA3 subfield of 3% dimethyl sulfoxide (DMSO), 6 nmol rosiglitazone (RGZ) or 500 ng GW9662. (A)** Qualitative analysis. **(B)** Quantitative analysis. Values in panel B are fold changes with reference to sham-control and are mean ± standard error of the mean (SEM) of four animals per experimental group. *P < 0.05 versus sham-control group, +*P* < 0.05 versus sham-control, DMSO + KA or GW9662 + KA group, and #*P* < 0.05 versus sham-control, DMSO + KA or RGZ + KA group in the Scheffé multiple-range test.

We have shown previously that an excessive oxidative and nitrosative stress followed by the release of cytochrome *c* to the cytosol that triggers the caspase cascades, leads to apoptotic cell death in the hippocampus during experimental status epilepticus [20,21]. Our final series of experiments explored whether the upregulated PPARγ/UCP2 signaling pathway plays a significant role in ameliorating this process. We found that whereas pretreatment with rosiglitazone (6 nmol) significantly reduced, the extent of Bax translocation from cytosol to mitochondria and cytochrome *c* translocation from mitochondria to cytosol in the CA3 areas 24 h after experimental temporal lobe status epilepticus, GW9662 (500 ng) significantly augmented it (Figure 8). Comparable results were obtained from qualitative (Figure 9A) and quantitative (Figure 9B) analysis of DNA fragmentation as another index for apoptosis, 7 days after the induction of status epilepticus.

## Discussion

Based on a clinically relevant animal model, the present study provided novel evidence to support an antioxidant role for UCP2 in temporal lobe status epilepticus. Specifically, our results revealed that upregulation of UCP2 expression induced by experimental status epileptics decreased oxidative stress, reduced mitochondrial dysfunction, blunted mitochondrial intrinsic apoptotic cell death pathway and protected against neuronal cell death in the hippocampal CA3 subfield.

PPARs are known to modulate the inflammatory and oxidative response [36]. The beneficial effects of PPARs in inflammatory diseases are exerted through regulation of cytokine production and adhesion molecule expression by interfering with transcription factors, including nuclear factor-κB (NF-κB), activator protein-1 (AP-1), signal transducers and activators of transcription (STATs) [36-38]. Treatments with PPARγ agonists increase the expression of UCP2 in both animal and cell studies [25,39,40], suggesting that UCP2 may be regulated by PPARγ activity. We have demonstrated previously [23,24] that the PPARγ agonist, rosiglitazone enhances UCP2 expression in the hippocampal neurons, leading to protection against oxidative stress and neuronal cell death associated with cerebral ischemia. We also showed previously [25] that gene knockdown of UCP2 by antisense oligonucleotide or pharmacological pretreatment with PPARγ agonist and antagonist also pointed to an antioxidative role for UCP2 in the brain stem against neurogenic hypertension. Moreover, in mice overexpressing human UCP-2 gene, brain damage was diminished after experimental stroke and traumatic brain injury, and neurological recovery was enhanced [18]. In rat cultured cortical neurons, overexpression of UCP-2 gene reduced cell death and inhibited caspase-3 activation induced by oxygen and glucose deprivation [18]. It is intriguing that results from the present study also showed that pretreatment with rosiglitazone increased mitochondrial UCP2 expression, reduced the extent of protein oxidation, O_2_^·-^ overproduction and dysfunction of mitochondrial respiratory enzyme complex I, hindered the translocation of Bax or cytochrome *c* between cytosol and mitochondria and reduced neuronal damage in the hippocampal CA3 subfield elicited by experimental status epilepticus. In contrast, treatment with the PPARγ antagonist, GW9662 exerted opposite effects. Thus, the present study provided a novel demonstration of an antioxidant role for the PPARγ/UCP2 signaling pathway against oxidative stress and mitochondrial dysfunctions that reduced neuronal cell injury in the hippocampal CA3 subfield after the experimental model of temporal lobe status epilepticus.

Neuroprotection following prolonged seizures, such as status epilepticus should encompass not only the prevention of neuronal cell death, but also preservation of neuronal and network function. Less well studied are the protective mechanisms elicited by seizure activity especially under status epilepticus. Except for the detrimental chain reaction under status epilepticus, acute response protein to counteract these detrimental effects may be elicited as an endogenous protective mechanism. Endogenous neuronal survival mechanisms following prolonged seizure insult are those that have been evolutionarily conserved and may trigger a number of signaling pathways to exert the protective effect and therefore be strong candidates to imply as therapeutic strategies [41]. In animal studies with status epilepticus, several endogenous protective mechanisms to lessen neuronal damage were proposed, including activation ERK1/2, epileptic tolerance, vascular endothelial growth factor, activation of adenosine A1 receptors, erythropoietin receptor [41-45]. Based on real-time PCR and western blot analyses, we demonstrated a significant increase in UCP2 mRNA in the hippocampal CA3 subfield after KA-elicited status epilepticus, followed by augmented UCP2 protein levels. In addition, immunofluorescence staining demonstrated that the activated UCP2 was mainly in the mitochondria of hippocampal CA3 neurons. Thus, our results suggested that mitochondrial UCP2 may play an endogenous neuroprotective role against hippocampal neuronal cell damage under the stress of prolonged epileptic seizures.

Several antioxidant systems are present in the cell to counteract oxidative stress and to restore redox balance, and may be considered endogenous protective mechanisms under pathological conditions. In addition to the documented ROS-detoxifying enzymes and low molecular weight antioxidants, whether mitochondrial UCP functions as a natural antioxidant defense mechanism against oxidative stress is still debatable. Mitochondrial UCPs control the leakage of protons across the inner mitochondrial membrane and have emerged as an important modulator for oxidative stress [17,46]. As UCP2 are most prevalent in the nervous system, and a majority of the neurodegenerative disorders engages free radical production [13,17,46], it is reasonable to propose that UCP2 induction will be involved in these neurological disorders, including status epilepticus. Although the physiological role of UCP2 is still not clear, emerging evidence suggests that UCP2 may be related to the regulation of mitochondrial membrane potential, regulation of ROS production, preservation of calcium homeostasis, modulation of neuronal activity, and inhibition of cellular damage and inflammation [17,46,47]. Several recent studies stressed the role of protective effects of UCP2 against the neuronal cell damage after cerebral ischemia and brain trauma [18,24,46-49]; limited studies explored the role of UCP2 in epileptic seizures. In transgenic mice that express UCP2 constitutively in the hippocampus, there is an attenuation of seizure-induced neuronal death and an increase in mitochondrial number and ATP levels, alongside a parallel decrease in free radical-induced damage [50]. Modulation of UCP2 expression and function by dietary fat protects neonatal rats against seizure-induced brain damage associated with oxidative stress and mitochondrial dysfunction [51]. In the present study, we found that mitochondrial UCP2 was significantly upregulated in the hippocampal CA3 region 12 to 48 h after the induction of experimental status epilepticus, at a time-point that lagged behind the increase in protein carbonylation and O_2_^·-^ These results indicate that the endogenous activation of mitochondrial UCP2 in hippocampal CA3 neurons under prolonged epileptic seizures may be a consequence of the increase in ROS production.

Mitochondrial dysfunction has been implicated as an important factor in the pathogenesis of seizure-induced neuronal cell death [2,10,52]. As the cellular powerhouse, the primary function of mitochondria is the production of cellular energy in the form of ATP by way of oxidative phosphorylation through the mitochondrial respiratory chain [53]. However, mitochondrial metabolism is also responsible for a majority of ROS production in cells and complex I of the mitochondrial electron transport chain is noticeably more susceptible to both oxidative and nitrosative stress than other respiratory chain complexes [54]. Dysfunction of complex I may lead to incomplete mitochondrial electron transport and reduced ATP production. We reported previously [5,19-21] that activation of NF-κB in hippocampal CA3 neurons upregulates NOS II gene expression, accompanied by an increase in O_2_^·-^ production and peroxynitrite formation, followed by reduction in mitochondrial complex I activity, leading to apoptotic neuronal cell death in the hippocampus via the intrinsic mitochondrial apoptotic pathway. Therefore, the dysfunction of complex I may be an important biochemical hallmark of seizure-induced neuronal cell death in the hippocampus and may play a crucial role in the mechanism of epileptogenesis [2,10]. It follows that our demonstration of an increase or decrease in mitochondrial UCP2 expression induced by rosiglitazone or GW9662 underlying the attenuation or exacerbation of seizure-induced mitochondrial complex I dysfunction, suggests that another cellular role for UCP2 induced by experimental status epilepticus is amelioration of bioenergetics inefficiency in the hippocampus. Thus, UCP-2 may be an inducible protein that provides a neuroprotective effect by activating cellular redox signaling as well as by inducing mild mitochondrial uncoupling [18].

One of the decisive steps of the apoptotic cascade is permeabilization of the outer mitochondrial membrane [55], which leads to the release of cytochrome *c* from the intermediate space, followed by the activation of caspase-dependent apoptotic signaling. It is generally contended that the anti-apoptotic members of the Bcl-2 family work to prevent cytochrome *c* release by stabilizing the mitochondrial membrane barrier function and the pro-apoptotic members tend to induce cytochrome *c* release by permeabilizing the mitochondrial membrane [55,56]. Translocation of Bax from the cytosol to mitochondria is induced during apoptosis, and this process is inhibited by Bcl-2 [30,57]. The evidence of Bcl-2 family involvement in seizure-induced neuronal cell death has been demonstrated in recent studies, and both pro-apoptotic and anti-apoptotic Bcl-2 family proteins were found to be activated by seizures [1,58]. However, conflicting results on the expressional regulation of Bcl-2 family proteins in seizure-induced neuronal cell death have emerged [1,58]. The reason for the discrepancy is currently not well elucidated, but may be related to the severity and duration of the disease conditions, or differences in the experimental methods employed. Increased Bax translocation from cytosol to mitochondria in the hippocampus has been reported in an animal model of KA-induced epileptic seizures [59]. Bax has been detected in clusters and accumulations on the outer surface of mitochondria in the hippocampal neurons after intra-amygdala KA-induced seizures [60]. In the present study, we observed that the progressive translocation of cytosolic Bax to the mitochondria, alongside an increase in cytosolic presence of cytochrome *c*, are indicative of an interplay between Bax and cytochrome *c*-dependent apoptotic cell death in the hippocampus following status epilepticus. Whereas Bcl-2 is reported to be upregulated during seizure-induced neuronal cell death [58], the expression of Bcl-2 did not show significant changes in our present study. This discrepancy may be related to the highly specific functions and subcellular locations of Bax and Bcl-2; Bax protein is found in both cytoplasmic and mitochondrial compartments, and Bcl-2 protein is largely mitochondrial [1,55,56]. The present study also provided novel results to suggest that upregulation of UCP2 in the hippocampus following experimental status epilepticus exerts its anti-apoptotic action by interacting with Bax mitochondrial translocation and downstream cytochrome *c*-dependent apoptotic cascades. Overexpression of UCP2 in transgenic mice ameliorated ischemia-induced Bcl-2 suppression in the brain [47]. In skin cancer cells, upregulation of UCP2 blocked p53 mitochondrial translocation, which regulates the pro-apoptotic effector Bax and reduced apoptosis during early tumor promotion [61]. Therefore, we suggest that the upregulated UCP2 in the hippocampus may prevent the mitochondrial translocation of Bax by stabilizing the inner mitochondrial membrane potential, resulting in an antagonism against the downstream apoptotic events under prolonged epileptic challenges.

Considerable controversy exists among reported models of seizure-induced damage with regards to the distribution, magnitude or form of neuronal cell death [1,2,22,62]. The nature of hippocampal neuronal cell death following prolonged seizure was reported to be either apoptotic, necrotic or both [1,22,62,63]. Programmed cell death mechanisms associated with cellular apoptosis have been shown to be activated after experimental status epilepticus [1,63]. Whereas CA3 neurons in the ipsilateral hippocampus exhibited a mild degree of necrosis or the intermediate forms of neuronal damage [22] that may be directly related to KA excitatotoxicity, our experimental model revealed that seizure-induced apoptotic cell death via cytochrome *c*/caspase-3-dependent signaling cascade was detected in the vulnerable CA3 neurons after a low dose of intrahippocampal administration of KA. We found that the degree of dysfunction of complex I respiratory chain enzyme was similar at 3 h and 24 h after experimental status epilepticus. This implied that the complex I dysfunction did not progress beyond 24 h in this animal model. In addition, our previous study found that preserved mitochondrial ultrastructural integrity and maintained energy metabolism 3 to 7 days following experimental status epilepticus is associated specifically with apoptotic, not necrotic, cell death in hippocampal CA3 neurons [22]. It follows that differences in animal models of seizures, variations in duration and intensity of the induced seizure activity, and metabolic disturbances after seizures are all contributing factors that determine the level of energy production in the mitochondria, leading eventually to diverse neuronal cell death fate in vulnerable regions of the hippocampus.

Our results showed a temporal decrease in PPARγ expression 6 h after experimental status epilepticus, followed by a significant increase of expression from 12 to 48 h in the hippocampal CA3 subfield. Whereas the design of the present study did not allow us to address the underlying mechanism, we are aware that a transient decrease in the expression of PPARγ protein under pathological conditions such as hypoxia, cerebral ischemia and interferon-γ or nerve growth factor treatment have been reported in neuronal and non-neuronal cells [38,64-66]. This effect may be attributed to the activation of ubiquitin-proteasome pathway or cytokines and inflammatory responses [38,64,67]. At the same time, transcription factors such as NF-κB, AP-1 and STATs are known to regulate cytokine gene expression and inflammatory response [68]. We have demonstrated previously [19] that significantly augmented nucleus-bound translocation of NF-κB and DNA binding activity of NF-κB in hippocampal CA3 neurons and glial cells occurs as early as 30 minutes after the elicitation of sustained seizure activity. Therefore, the transient decrease of PPARγ expression in the hippocampus during experimental status epilepticus may be related to activation of NF-κB and other inflammatory responses. However, the interrelationship between NF-κB and PPARγ in this experimental paradigm warrants further exploration.

## Conclusions

We demonstrated that activation of PPARγ upregulated mitochondrial UCP2 expression, which decreased overproduction of ROS, improved mitochondrial complex I dysfunction, inhibited mitochondrial translocation of Bax and prevented cytosolic release of cytochrome *c* by stabilizing the mitochondrial transmembrane potential, leading to amelioration of apoptotic neuronal cell death in the hippocampus following status epilepticus. These findings may offer a new vista in the development of more effective strategies to enhance this endogenous protective mechanism and reduce brain damage caused by status epilepticus.

## Abbreviations

ANOVA, one-way analysis of variance; AP-1, activator protein-1; BCA, bicinchoninic acid; CNS, central nervous system; COX IV, cytochrome c oxidase subunit IV; DMSO, dimethyl sulfoxide; DNP-hydrazone, 2,4-dinitrophenylhydrazone; ELISA, enzyme-linked immunosorbent assay; GAPDH, glyceraldehyde-3-phosphate dehydrogenase; GFAP, glial fibrillary acidic protein; hEEG, hippocampal electroencephalography; ip, intraperitoneal; KA, kainic acid; NADH, nicotinamide adenine dinucleotide; NF-κB, nuclear factor-κB; NO, nitric oxide; NOS II, nitric oxide synthase II; O2·-, superoxide anion; PBS, phosphate buffered saline; PCR, polymerase chain reaction; PPARγ, peroxisome proliferator-activated receptor γ; RT, reverse transcriptase; ROS, reactive oxygen species; SOD, superoxide dismutase; STATs, signal transducers and activators of transcription; UCP, uncoupling protein.

## Competing interest

The authors have no competing interests to declare in relation to this study.

## Authors’ contributions

YCC, SDC, AYWC and SHHC conceived and designed the experiments. YCC, TKL, SDC, HYH and CWL performed the experiments. YCC, HYH, SDC, AYWC and SHHC analyzed the data. YCC, TKL, SDC and SHHC wrote the paper. YCC, SDC and SHHC supervised the research. YCC, TKL, HYH, WNC, SDC and CWL collected data. All authors have read and approved the final version of this manuscript.

## Authors' informations

YCC is Professor of Neurology and Head of the Division of Epilepsy in Kaohsiung Chang Gung Memorial Hospital. TKL, WNC, CWL and SDC are Associate Professors of Neurology in Kaohsiung Chang Gung Memorial Hospital. HYH is Professor of Pathology and Head of Pathology in Kaohsiung Chang Gung Memorial Hospital. AYWC is Professor of the Center in Translational Research in Biomedical Sciences in Kaohsiung Chang Gung Memorial Hospital. SHHC is the National Chair Professor in Neuroscience, awarded by the Ministry of Education, Taiwan. He is also a Distinguished Chair Professor of Translational Medicine, and the founding and current director of the Center in Translational Research in Biomedical Sciences in Kaohsiung Chang Gung Memorial Hospital. SHHC is currently the President of the Federation of Asian and Pacific Pharmacological Societies.
